# Vaccinia Virus Immunomodulator A46: A Lipid and Protein-Binding Scaffold for Sequestering Host TIR-Domain Proteins

**DOI:** 10.1371/journal.ppat.1006079

**Published:** 2016-12-14

**Authors:** Sofiya Fedosyuk, Gustavo Arruda Bezerra, Katharina Radakovics, Terry K. Smith, Massimo Sammito, Nina Bobik, Adam Round, Lynn F. Ten Eyck, Kristina Djinović-Carugo, Isabel Usón, Tim Skern

**Affiliations:** 1 Max F. Perutz Laboratories, Medical University of Vienna, Vienna Biocenter, Dr. Bohr-Gasse 9/3, Vienna, Austria; 2 Biomedical Sciences Research Complex, University of St. Andrews, North Haugh, St. Andrews, Fife Scotland, United Kingdom; 3 Structural Biology, IBMB-CSIC, Baldiri Reixach, 13–15, Barcelona, Spain; 4 Georg August University of Göttingen, Department of Structural Chemistry, Tammannstr. 4, Göttingen, Germany; 5 European Molecular Biology Laboratory, Grenoble Outstation, 71 Avenue des Martyrs, CS, Grenoble, France; 6 European XFEL GmbH, Notkestraße 85, Hamburg, Germany; 7 San Diego Supercomputer Center, University of California, San Diego, La Jolla, California, United States of America; 8 Department of Chemistry and Biochemistry, University of California at San Diego, La Jolla, California, United States of America; 9 Max F. Perutz Laboratories, University of Vienna, Vienna Biocenter, Dept. of Structural and Computational Biology, Campus Vienna Biocenter 5, Vienna, Austria; 10 Department of Biochemistry, Faculty of Chemistry and Chemical Technology, University of Ljubljana, Aškerčeva 5, Ljubljana, Slovenia; 11 ICREA, Pg. Lluis Companys 23, Barcelona, Spain; Washington University, UNITED STATES

## Abstract

Vaccinia virus interferes with early events of the activation pathway of the transcriptional factor NF-kB by binding to numerous host TIR-domain containing adaptor proteins. We have previously determined the X-ray structure of the A46 C-terminal domain; however, the structure and function of the A46 N-terminal domain and its relationship to the C-terminal domain have remained unclear. Here, we biophysically characterize residues 1–83 of the N-terminal domain of A46 and present the X-ray structure at 1.55 Å. Crystallographic phases were obtained by a recently developed *ab initio* method entitled ARCIMBOLDO_BORGES that employs tertiary structure libraries extracted from the Protein Data Bank; data analysis revealed an all β-sheet structure. This is the first such structure solved by this method which should be applicable to any protein composed entirely of β-sheets. The A46(1–83) structure itself is a β-sandwich containing a co-purified molecule of myristic acid inside a hydrophobic pocket and represents a previously unknown lipid-binding fold. Mass spectrometry analysis confirmed the presence of long-chain fatty acids in both N-terminal and full-length A46; mutation of the hydrophobic pocket reduced the lipid content. Using a combination of high resolution X-ray structures of the N- and C-terminal domains and SAXS analysis of full-length protein A46(1–240), we present here a structural model of A46 in a tetrameric assembly. Integrating affinity measurements and structural data, we propose how A46 simultaneously interferes with several TIR-domain containing proteins to inhibit NF-κB activation and postulate that A46 employs a bipartite binding arrangement to sequester the host immune adaptors TRAM and MyD88.

## Author Summary

Viruses possess mechanisms to interfere with the host immune system to enhance their replication. Vaccinia virus, the viral vaccine used to eradicate smallpox, synthesizes many such proteins. The vaccinia virus protein A46 is one of a series of proteins preventing expression of host proteins that induce an anti-viral state. A46 acts early to inhibit anti-viral state induction by specifically binding to certain host adapter proteins such as MyD88 and TRAM. Here, we extend our knowledge of the A46 structure by determining the structure of the protein's N-terminal domain to be an unusual lipid binding fold. In addition, the full-length A46 molecule has a novel quaternary structure that can both bind proteins and lipids, indicating that A46 uses a variety of interactions to sequester host proteins, thus impairing the activation of the anti-viral state and improving the efficiency of viral replication.

## Introduction

Viral infection depends not only on the rate and precision of viral reproduction, but also requires a simultaneously efficient inhibition of host immune responses. Viruses have evolved varied strategies to interfere with immune responses of the host, including production of secreted molecules that mimic innate immune receptors, molecules that trap cytokines as well as the shut-off of the cellular transcription and translation machinery [1, 2]. Vaccinia virus (VACV), the virus used to eradicate smallpox, has been extensively studied as a model of virus-host interaction because of its plethora of anti-immune strategies and its large arsenal of immunomodulator tools [3]. Further interest in VACV stems from its role as a vaccine vector against important infectious diseases and its potential role against cancer [4, 5].

Amongst approximately 200 genes in the VACV genome, only half encodes for the viral replication machinery; many of the remaining gene products have roles as extra- and intracellular modulators of the host immunity [6]. The VACV intracellular immunomodulators form a family of Bcl-2-like (B-cell lymphoma 2 like) proteins with low sequence identity but high structural similarity to the eukaryotic Bcl-2 protein family [7]. Eukaryotic Bcl-2 proteins present a diverse group of pro- and anti-apoptotic regulators that share α-helical BH domains [3, 8]. To date, 11 Bcl-2-like proteins encoded by VACV have been identified. Those such as A46, A49, A52, B14, N1, K7 and F1 have an experimentally confirmed Bcl-2 fold [9–16]; others such as C1, C6, C16/B22 and N2 are predicted to have such a fold [10, 17, 18].

NF-κB is a transcriptional factor that responds to the stimulation of Toll-like-receptors (TLRs) and Interleukin-like-receptors (IL-1R) by inducing expression of effector molecules. In the uninfected cell, inactive NF-κB is located in the cytoplasm as a precursor or in a complex with its inhibitor (IκB). Upon stimulation of TLRs by pathogens, a signaling cascade is initiated through the recruitment of adaptor proteins (e.g. MyD88, MAL/TIRAP, TRIF, TRAM) by the cytoplasmic domains of TLRs, consequent stepwise activation of IRAK2-IRAK6-IRAK4 kinases followed by activation of TRAF6 ubiquitin ligase and activation of the IKK (IκB kinase) complex. Finally, the release of the active form of NF-κB results from processing of the precursors or degradation of IκB. Nuclear migration of the free NF-κB permits expression of a range of cytokines allowing the development of both innate and adaptive immune responses [19]. VACV Bcl-2-like immunomodulators disrupt NF-κB activation pathways at different stages by targeting various components [3, 7]. The A46 protein acts close to the plasma membrane by binding numerous TIR-domain containing adaptor proteins such as MyD88, MAL/TIRAP, TRAM and TRIF as well as TLR4 to prohibit further signal propagation [20].

We recently determined the structure of the Bcl-2 domain of A46 comprising residues 87–229 [9]. However, structural information on the N-terminal domain (residues 1–86), its position relative to the Bcl-2-like domain and a plausible function were lacking. Here, we report the crystal structure of the A46 N-terminal domain comprising residues 1 to 76 and demonstrate that this domain binds fatty acids. Further, small-angle X-ray scattering (SAXS) was employed to derive a structural model of full-length of A46(1–240). Using a SAXS-derived model of A46 together with biochemical data, we postulate a mechanism explaining the biological function of this unusual VACV immunomodulator protein.

## Results

### Comparison of the members of VACV Bcl-2-like proteins

**Fig 1:**
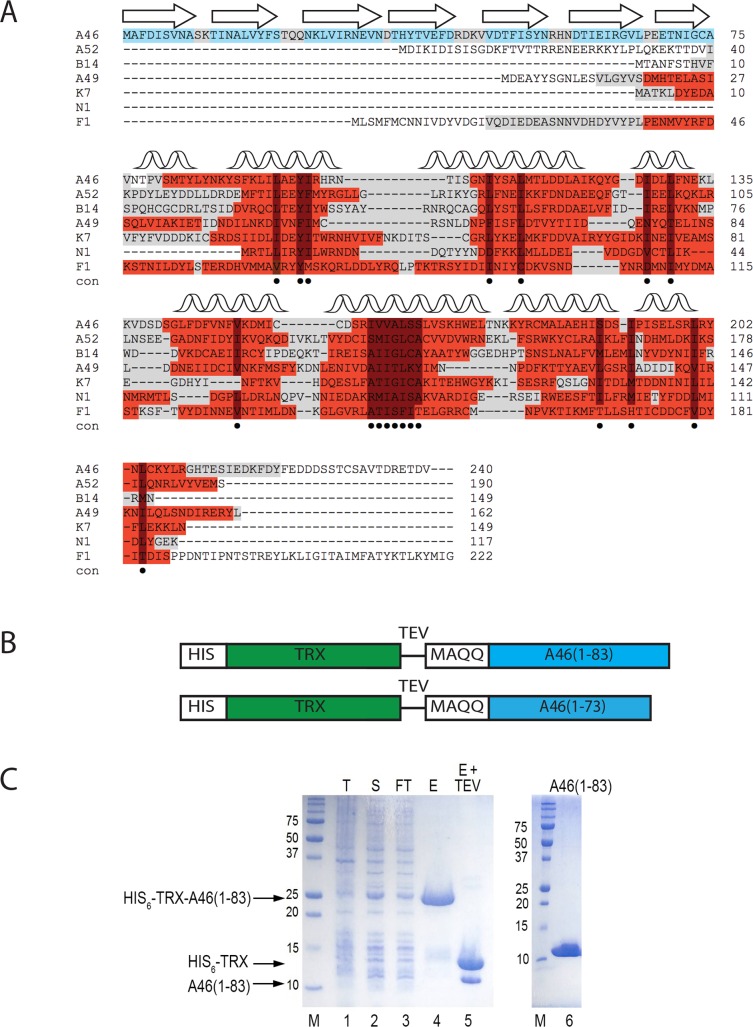
The VACV A46 protein constructs and purification of A46(1–83). A, Structural alignment of VACV Bcl-2-like immunomodulators. The structural alignment was generated using the T-coffee online algorithm [22] with additional manual correction. Protein Data Bank (PDB) codes are 4LQK, 4M0S (A46, [9, 23]), 2VVW (A52, [10]), 2VVY (B14, [10]), 4D5S (A49, [11]), 3JRV (K7, [14]), 2I39 (N1, [12]), 2VTY, 4D2L (F1, [15, 16]). Bullets indicate the residues forming the hydrophobic core of the Bcl-2-like domains [7]. Portions of proteins seen in three dimensional protein structures are highlighted in grey, helices in red and β-strands in cyan. B, Schematic representation of expression constructs for the A46 N-terminal domain. HIS, hexahistidine tag. TRX, thioredoxin solubilisation tag (green). TEV, TEV protease cleavage site. A46 variants (light blue). C, SDS-PAGE analysis of the purification of A46(1–83). Lane 1, crude cell extract (T); lane 2, total soluble protein in crude cell extract loaded on to Ni-NTA beads (S); lane 3, flow-through from Ni-NTA beads (FT); lane 4, eluate from Ni-NTA beads with imidazole (E); lane 5, eluate after incubation with TEV protease (E+TEV); lane 6, 15 μg of final product from concentrated pooled fraction after SEC. The gel contained 15% acrylamide; proteins were visualized with Coomassie Brilliant Blue R250. Apparent molecular sizes (in kDa) are indicated on the left.

Members of the VACV Bcl-2-like family whose structure has been determined mainly comprise a single Bcl-2-like domain with an N- or a C-terminal extension (ranging from 5 to 80 amino acids) or both (Fig 1A). At present, structural information is only available for the Bcl-2-like domains and a short unstructured N-terminal region of F1L [21] but not for the rest of extensions. However, the N-terminal extension of A46 spanning residues 1–80 was predicted by PSIPRED (31) to comprise exclusively β-strands. Previous studies using limited proteolysis on the full-length A46 protein confirmed the presence of a structured N-terminal domain in the first 80 residues, suggesting that it would be amenable to crystallography (Fig 1A) [9].

### Expression and characterization of the N-terminal domain of A46

To examine the structure and function of the N-terminal domain of A46, we designed two constructs for expression in *E*. *coli*. Both protein expression constructs contained the first methionine of the full-length protein and comprised 73 or 83 A46 residues, as constructs with fewer than 73 residues were either insoluble when His_6_-tagged or could not be removed from the MBP expression tag. Both variants contained an additional four amino acids (MAQQ, Fig 1B) to improve solubility as observed with full-length A46 [9]. Thus, both fusion proteins had the following structure: His_6_-TRX-TEVsite-MAQQ-A46(1-73/83). The average yield of both proteins was approximately 2.5 mg highly purified protein per L of bacterial culture. However, as we only obtained diffraction quality crystals with A46(1–83), we performed all subsequent work with this variant (Fig 1C).

### A46(1–83) selectively binds TIR/MyD88 but not TIR/MAL or TIR/TRAM binding partners

We first examined the ability of A46(1–83) to bind the TIR domains of its proposed cellular binding partners such as MyD88 and MAL. Using microscale thermophoresis, we previously demonstrated that the C-terminal domain of A46 binds in the low micromolar range to these TIR domains; the K_D_ values were slightly lower than those observed with the full-length protein (Table 1) [9]. In contrast, the N-terminal A46(1–83) binds to TIR/MyD88 but not to TIR/MAL. The K_D_ value was 8.8 μM, compared to that of 0.52 μM for A46(1–229). We also examined the binding of the TIR/TRAM domain, another proposed A46 *in vivo* binding partner, to A46 [20, 24]. The interaction of full-length A46 and its C-terminal domain with TIR/TRAM shows K_D_ values of 2.39 μM and 3.62 μM, respectively. However, under the conditions used, A46(1–83) did not bind to TIR/TRAM (Table 1).

**Table 1:** Analysis of binding of A46 variants to TIR domains of MyD88, MAL and TRAM (K_D_, μM).

	Quaternary Structure	MyD88	MAL	TRAM
A46(1–229)	tetramer	0.46*, 0.52*	1.44*, 1.56*	2.39 ± 0.16
A46(87–229)	dimer	0.89*, 1.24*	2.24*, 2.30*	3.62 ± 0.52
A46(1–83)	tetramer	8.80 ± 0.63	N.D.	N.D.

The values marked with asterisk (*) were previously reported [9] and are shown here for comparison. The remaining values are presented as an average from at least three measurements ± standard error of the mean. N.D., not detected.

### A46(1–83) is unable to perform an immunomodulatory function *in vivo*

**Fig 2:**
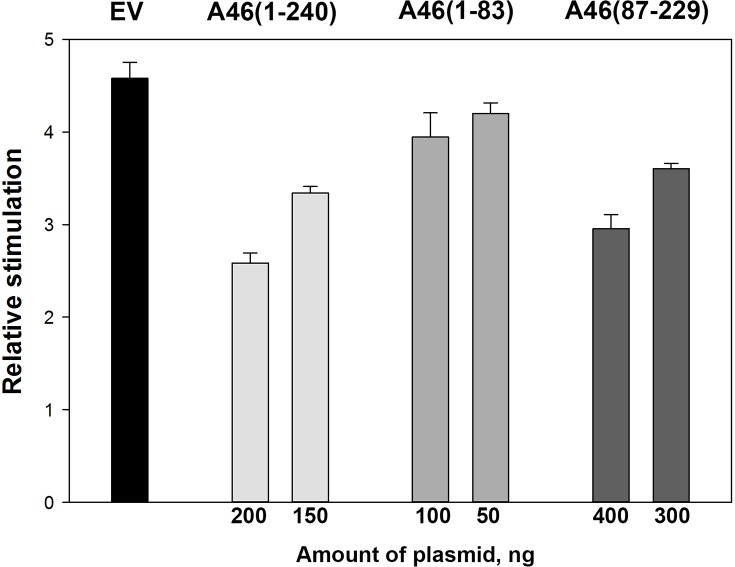
Inhibitory effect of full-length and truncated constructs of A46 on the IL-1β stimulated NK-κB transcription. HEK293T cells were transfected with the indicated amounts of plasmids. Plasmid amounts were adjusted so that approximately the same amounts of each A46 variant were expressed. After 40 h of incubation, cells were stimulated with 0.32 ng/ml of IL-1β and incubated for a further 6 h. NF-κB reporter gene activity was then measured. Data are expressed as the relative stimulation from a representative experiment from a minimum of three separate experiments, each performed in triplicate. Error bars represent the standard deviation from the mean. EV, empty vector.

Given the binding of A46(1–83) to MyD88, we next examined whether this fragment was sufficient to prevent IL-1β induction of NF-κB-mediated transcription using similar cell-based assays to those described previously [9]. Plasmid amounts were adjusted so that approximately the same amounts of each A46 variant were expressed; the total amount of transfected DNA (500 ng) was kept constant by the addition of empty pCAGGS vector. Unlike full-length A46 and the truncated variant A46(87–229), the N-terminal domain exhibits no appreciable inhibition of the IL-1β driven induction of NF-κB (Fig 2; see figure legend for statistics). Thus, binding of A46 residues 1–83 is insufficient to independently fulfil an immunomodulatory role.

### *Ab initio* solution of crystallographic phases

We initiated structural studies of the functional form of the N-terminal domain of A46(1–83) by setting up crystallization trials with commercial screens. Small single crystals of around 20 μm in size were observed after 1 week of incubation at 22°C. They failed, however, to grow larger; nevertheless, several datasets were collected using the beam line for high throughput macromolecular data-collection MASSIF at ESRF, rendering the highest resolution between 1.8 and 2.3 Å. With no known close homologue in the PDB database, we were unable to solve the phase problem by molecular replacement. Thus, we labelled the protein with selenomethionine; diffraction quality crystals grew in the conditions used for the native protein. Data sets for SAD were collected using the MASSIF beamline up to 1.55 Å resolution. However, we were unable to phase the structure using the anomalous signals, most likely because all three methionines in the protein lie in the very N- and C-termini of the A46(1–83) construct and, consequently, are located in flexible regions. Finally, the phases were obtained by ARCIMBOLDO_BORGES [25] crystallographic software. The program exploits tertiary structure libraries extracted from the Protein Data Bank for *ab initio* phasing. A library of 7650 superimposed polyalanine models, representing 925300 variations on the fold of three stranded antiparallel β-sheets totalling 20 amino acids, was used as fragment hypotheses. This three strands arrangement is most frequently found in β-sheets. Computations were executed on the Gordon supercomputer at the San Diego Supercomputer Center in California. A partial solution was obtained upon location with PHASER [26] of 4 models extracted from the unrelated PDB structures 2QLG, 2GSK, 2EFU, 4DCB as indicated by SHELXE [27] trace correlation coefficients above 40%. The root mean square deviation (rmsd) of the solving library models against the final structure was in the range of 0.35 Å (model from 4DCB) and 0.61 Å (model from 2EFU).

### Crystal structure of A46(1–83)

**Fig 3:**
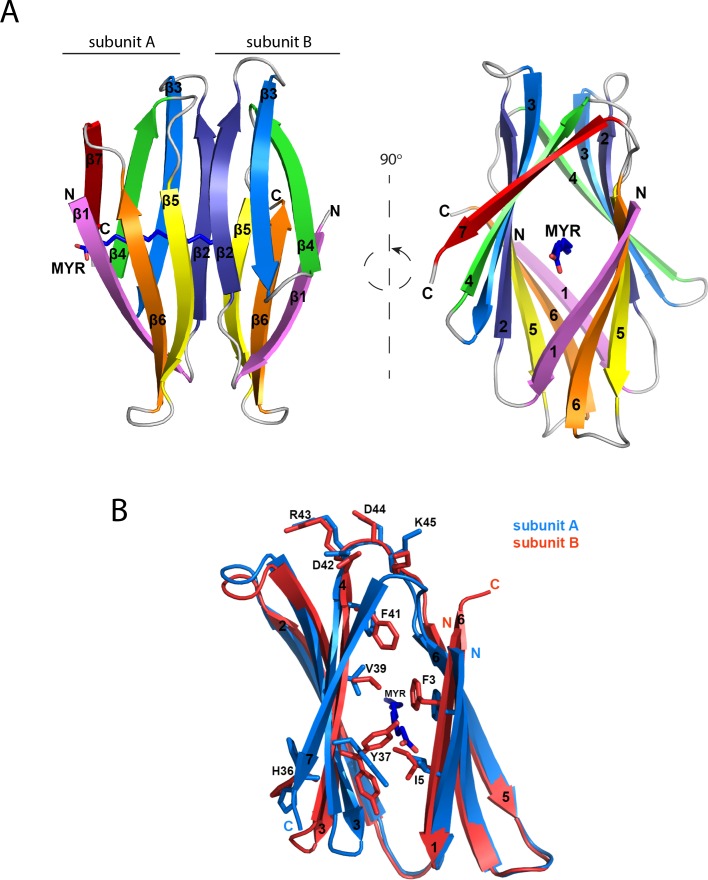
Overall structure of the N-terminal domain of A46. A, Two views of the structure of A46(1–83). The dimer comprises subunit A (7 β-strands) and subunit B (6 β-strands) with the subunits coloured as rainbows from the N- to the C-termini. Myristic acid co-crystallized as a ligand inside of the cavity and is depicted as sticks; carbon atoms are blue and oxygen ones are red. B, Superimposition of subunit B on subunit A of A46(1–83) dimer. Subunit A (in blue) and subunit B (in red) were superimposed in PyMOL. MYR, myristic acid. The amino acids most differing within subunits are indicated. Panel B is related to panel A by a counter-clockwise rotation of 30°. Drawings were made using PyMOL [29].

A46(1–83) crystallized with two molecules in the asymmetric unit; electron density for a bound ligand, later identified as myristic acid, was found inside one of the molecules. The two A46 molecules comprise two β-sheets arranged head to head as an extended β-sandwich (Fig 3A, Table 2). A tetramer is formed over a crystallographic twofold axis continuing the β-sandwich with the second dimer rotated approximately 90° relative to the first (S1A and S1B Fig). The PISA server [28] estimates both association interfaces to be present in solution, burying 1276 and 941 Å^2^.

**Table 2:** Crystallographic data

**DATA COLLECTION**	
Source	MASSIF-1, ESRF
Wavelength (Å)	0.9793
Resolution (Å)	41.84–1.55 (1.65–1.55)*
Space group	C2
Unit cell (Å, °)	a = 65.79, b = 59.58, c = 47.26 α = 90, β = 117.7, γ = 90
Molecules / a.u.	2
Unique reflections	23425 (3973)*
Completeness (%)	99.6 (98.9)*
CC_1/2_	100% (58%)*
R_merge_	0.036 (0.540)*
R_sigma_	0.032 (0.603)*
Redundancy	1.94 (1.93)*
<I/σ_I_>	13.0 (1.5)*
**REFINEMENT**	
R_work_/ R_free_	20.1/24.2
R.m.s.d. bonds (Å)	0.006
R.m.s.d. angles (°)	1.02
Ramachandran plot (%)	
favored/allowed/outliers	97.97/2.03/0

* Values in parentheses are for the highest resolution shell.

The A and B independent subunits show marked differences, with a C_α_ rmsd of 1.3 Å for the 45 common β-strand residues (Fig 3B); the tetramer can be described as an A/B/B/A arrangement. Subunit A has 7 β-strands whereas subunit B presents only 6, lacking the most C-terminal one (Fig 3A). No electron density is seen for either residues 77–83 in subunit A or 67–83 in subunit B, suggesting that these regions may constitute a flexible linker between N- and C-terminal domains in the full-length molecule.

### A46(1–83) and full-length A46 bind long-chain fatty acids

**Fig 4:**
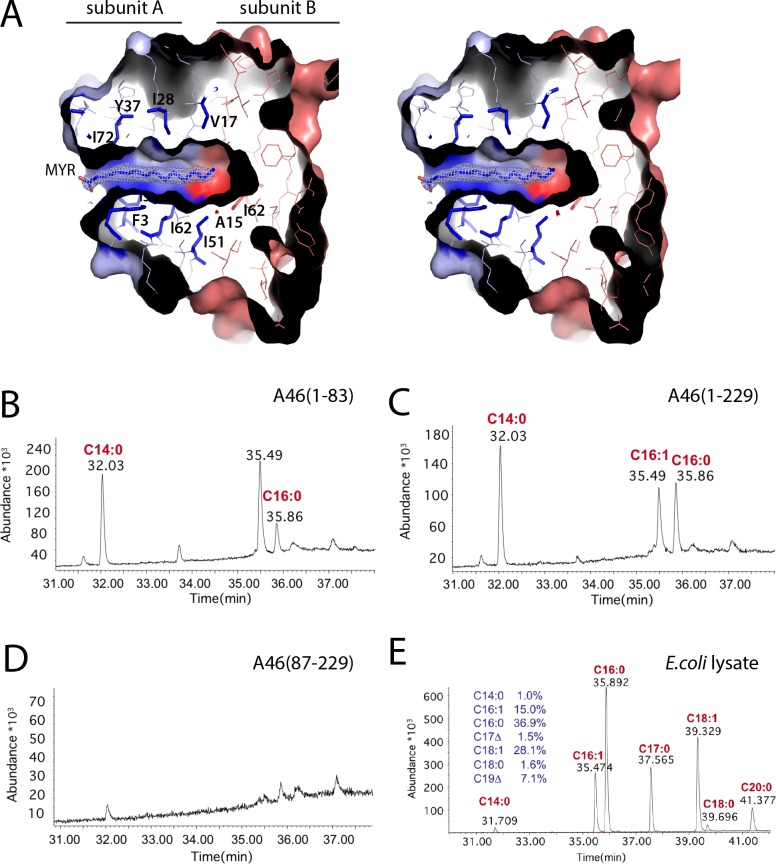
Identification of the fatty acids co-purified with A46 constructs. A, Stereo view of myristic acid accommodated in a hydrophobic cavity of A46(1–83) dimer. *2F*_*0*_*-F*_*c*_ electron density map of myristic acid contoured at 1σ. Subunit A is blue, subunit B is red. Myristic acid is depicted as sticks, hydrophobic residues are coloured in the colour of respective subunit. Drawings were made using PyMOL [29]. B, C, D Total fatty acid content of fatty acids present in the extract from A46(1–83), A46(1–229) or A46(87–229), respectively. Fatty acids released by base and converted to methyl esters and analysed by GC-MS (see material and methods for details). The figure shows the TIC chromatogram 31–38 min in which the identified fatty acid methyl ester species are marked. E, Typical content of fatty acid extract from *E*.*coli* DE3 cells grown in LB medium at 25°C.

A striking feature of the external β1-β7 face of the A subunit is a partially hydrophobic tunnel, spanning the whole subunit A and reaching into subunit B (Fig 4A). A length of 22 Å, an average radius of 2.5–3 Å and an overall cavity volume of 1150 Å^3^ (S2 Fig) were calculated with the software MOLE 2.0 [30, 31]. The tunnel is occupied by an extended well-defined electron density, reminiscent of a myristic acid molecule (Fig 4A). The omit electron density map for the ligand is presented in S3 Fig. Mass spectrometry and gas chromatography (GC) analysis of the lipids extracted from purified protein identified the fatty acids C14:0, C16:0 and C16:1 in complex with A46(1–83) (Fig 4B). Repetition of the experiment with a separate A46(1–83) preparation revealed the same three fatty acids but in different ratios, indicating that the relative amounts may be preparation dependent. However, in all preparations so far examined, the C14:0 fatty acid was highly enriched compared to its overall representation in *E*.*coli* cells (Fig 4E).

Further, the lipid extraction and identification by mass spectrometry was also done with three independently purified samples of the full-length A46 as well as two purified samples containing the C-terminal domain of A46 (87–229). For the full-length A46, we also identified the three co-purified fatty acids, C14:0, C16:0 and C16:1; in contrast, purified A46(87–229) lacked any complexed lipids (Fig 4C and 4D). Hence, only samples containing the N-terminal domain of A46, either purified independently or as a part of the full-length protein, are capable of binding fatty acids.

Subunit B, being partially penetrated by the fatty acid, cannot therefore lodge a second molecule. The cavity, present in subunit A, is collapsed in subunit B, bringing both β-sheets 3.5Å nearer (Fig 3B). Tyr37 adopts a dual conformation in the two subunits, suggesting a gate-keeper role as it folds back in subunit A to make room for the myristic acid (Fig 3B). The side chain of the preceding His36, pointing to the outside and located in the loop displaying highest differences between both subunits, also has two conformations. A single loop at each side of the sandwich joins both sheets, allowing the displayed flexibility. One loop (β1 to β2) is unchanged; the other (β4 to β5), containing four charged residues DRDK, differs between the subunits, altering its hydrogen bond pattern (Fig 3B). Together with His36, these electrostatic interactions may provide a lever for myristic acid binding. The absence of bound fatty acid as well as the lack of the β7 strand in subunit B results in a quite different interaction interface to that in subunit A, allowing association of two B subunits, with β1 occupying the position vacated by β7, and thus the assembly of the symmetric tetramer (S1 Fig).

**Fig 5:**
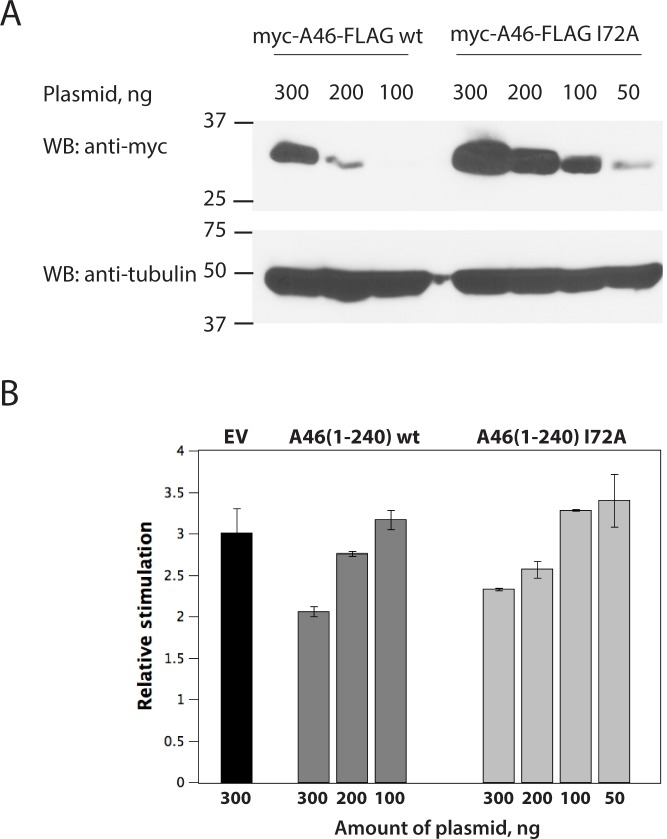
A46 I72A is impaired in the inhibition of TLR4-stimulated NK-κB transcription. A, Expression levels of the myc and FLAG-tagged A46 wild-type and I72A mutant in HEK293T cells. The cells were transfected with the indicated amounts of plasmids and the expressed A46 was detected by western blotting with anti-myc antibodies. ϒ-tubulin was used as a loading control and detected with anti-tubulin antibodies. B, Dose-dependent inhibition of the TLR4-stimulated NK-κB transcription by either A46 wild-type or I72A mutant. HEK293-TLR4 cells were transfected with the indicated amounts of plasmids. After 40 h of incubation, cells were stimulated with 500 ng/mL of LPS and MD2 supernatant and incubated for a further 7 h. NF-κB reporter gene activity was then measured. Data are expressed as the relative stimulation from a representative experiment from a minimum of two separate experiments, each performed in triplicate. Error bars represent the standard deviation from the mean. EV, empty vector.

We examined the lipid-binding properties of A46(1–83) by structure-based site-directed mutagenesis. We introduced the single mutations F3D, H36L, Y37A, Y37W, I72A into the expression plasmid for A46(1–83) and successfully expressed and purified protein from all variants. Analysis of their lipid content showed that all variants contained C14:0, C16:0 and C16:1 fatty acids. Furthermore, only the variant Y37A had a wild-type amount of lipids; all of the others had less bound lipid than the wild-type, with the variant I72A having the lowest value of 29% (S1 Table). To investigate whether the level of bound lipids influence the function of A46, the I72A mutant of full-length A46 was examined in a NF-κB transcriptional assay in TLR4-expressing HEK293 cells. The A46 I72A mutant was reproducibly expressed at higher levels, both in mammalian cells (Fig 5A) and bacteria. The A46 I72A variant could achieve similar levels of inhibition of NF-κB mediated signalling as the wild-type (Fig 5B); however, this level could only be reached when a 2-to-3-fold excess of A46 I72A was expressed compared to the wild-type variant (Fig 5A and 5B). Thus, the lower lipid binding capacity of A46 I72A impairs its ability to inhibit TLR4 signalling.

### A46 is a tetramer in solution

**Fig 6:**
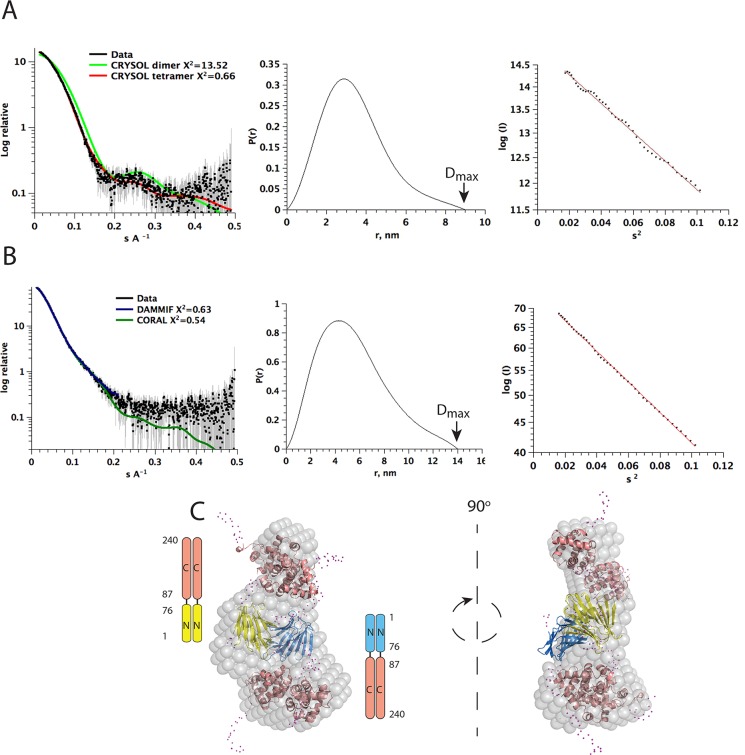
SAXS analysis of the N-terminal domain of A46(1–83) and full-length A46(1–240). A, SAXS analysis of the N-terminal domain of A46 (1–83). Experimental scattering profile is shown with black dots, theoretical scattering curve of A46(1–83) tetramer formed by a dimer in the asymmetric unit and its symmetry mate is in red and the one from the asymmetric unit dimer in green. The graph representing P(r) function with indicated D_max_ is in the center and Guinier plot on the right. B, SAXS analysis of the full-length A46(1–240). Experimental scattering profile is shown with black dots, calculated scattering curves from SAXS models are presented in blue (*ab initio* model calculated by DAMMIF) and in green (rigid body modeling by CORAL). The graph representing P(r) function with indicated D_max_ is in the center and Guinier plot on the right. C, Superimposition of the *ab initio* model envelope (grey surface) with the rigid-body modeling of the N- and C-terminal domains. The N-terminal domain (1–83) is rendered in yellow and blue, with one dimer is yellow and the other one is blue. The C-terminal domains (87–229) are rendered in salmon. The flexible regions are presented as magenta dots and include N-termini of A46(1–83), C-termini of A46(87–229) and the linker between A46(1–83) and A46(87–229).

In the light of the crystal structure, we analysed the oligomeric state of A46(1–83) using SAXS (Table 3). The theoretical scattering curve of the A46(1–83) tetramer in crystals presents a good fit to experimental data with Chi^2^ (Crysol [32]) of 0.66 (Fig 6A) versus a very poor fit for the possible dimer found in the asymmetric unit with Crysol Chi^2^ of 13.52 (Fig 6A).

**Table 3:** SAXS data

	A46(1–83)	A46(1–240)
**DATA COLLECTION PARAMETERS**		
Instrument	BioSAXS BM29, ESRF	
Beam geometry (μm)	700 x 700	
Wavelength (keV)	12.5	
q range (Å-1)	0.04–0.50	
Exposure time (sec)	10 frames each 1 second	
Concentration range (mg ml -1)	15.50	4.40
Temperature (K)	293	
**STRUCTURAL PARAMETERS**		
I(0) (cm-1) from Guinier	14.91 ± 0.02	75.53 ± 0.11
Rg (Å) (from Guiner)	2.61 ± 0.09	4.27 ± 0.08
Volume (Å^3) from Porod	68000	199000
**MOLECULAR MASS DETERMINATION**		
Mr estimate from volume (Da)	38857	113714
Calculated monomeric Mr from the sequence (Da)	10012	28221
**SOFTWARE EMPLOYED**		
Primary data reduction	EDNA pipeline [33]	
Data processing	PRIMUS	
Rigid body modeling	CORAL	
3D representation	PyMOL	
Graphics representation	QtiPlot	

How are the N-terminal and C-terminal domains of A46 oriented relative to one another? To address this question, we performed SAXS experiments on full-length A46(1–240) (Fig 6B, Table 3). The envelope is shown in Fig 6C, together with the fitting of the N- and C-terminal structures. This arrangement agrees with the tetrameric nature of the A46(1–240) and with proteinase digestion of the linker leading to the production of two domains with almost all proteinases tested [9].

## Discussion

We have determined the first structure of a structured N-terminal extension of a VACV Bcl-2-like immunomodulator; additionally, we also show that it is complexed with myristic acid. The A46(1–83) domain crystallized, forming regular continuous strands in a simple β-sandwich structure with few disordered residues (Fig 3A; S1 Fig). Nevertheless, the solution of the X-ray structure was complex. Due to the crystals' small size, automatic beam focussing at the MASSIF beam line was essential. Additionally, selenium anomalous signals could not be used because of the position of the methionine residues. Molecular replacement also failed due to lack of a known protein structure to be used as search model. However, the regular crystal packing allowed high-resolution data sets to be obtained that were initially processed at 1.55 Å resolution. This high resolution data, together with the short length of the protein, allowed the phases to be solved using *ab initio* methods [25]. In this method, which has been used successfully for numerous α-helical structures [34], fragments of known structures are employed as small search models. In our study, phases could be solved by a protein fragment of three β-strands that resembles part of the structure of A46(1–83), revealing two molecules in the asymmetric unit.

Refinement of the structure allowed the determination of electron density for residues 1–76 of subunit A and 1–66 in subunit B. The electron density showed clearly that both subunit A and subunit B were comprised entirely of β-sheets, confirming previous bioinformatic predictions that the N-terminus of A46 has a β-sheet arrangement. Unexpectedly, in the subunit B, the C-terminal strand β7 is disordered and not visible in the electron density. We propose that this difference allows A46(1–83) to form tetramers via the subunit B interfaces whilst interacting with ligands through the subunit A interfaces.

A further wholly unexpected feature of A46(1–83) is a partially hydrophobic cavity which spans the entire subunit A and part of subunit B. The cavity is open on the side of the A interface and accommodates long chain fatty acids that were co-purified from the *E*.*coli* cell lysate. In the X-ray structure of A46(1–83), clear electron density for C14:0 myristic acid was found (Fig 4A), with the hydrophobic tail buried in the cavity whereas the carboxyl group is open to the solvent. Such an orientation leads us to hypothesize that the cavity might serve as a specific binding pocket for myristoylated binding partners. To this end, TRAM is the only binding partner of A46 known to be myristoylated; myristoylation is indeed essential for its innate immune function, providing correct location of TRAM to the membranes [35]. Binding of A46 to the myristate of TRAM would prevent the insertion of TRAM into the membrane and thus circumvent intracellular signalling. An acceptable alternative hypothesis would be that the bound fatty acids induce asymmetry of the A46(1–83) dimer, as they block a polymerization interface equivalent to B/B and prevent binding of a second fatty acid copy in subunit B. In such manner, using the same primary sequence, a dimer of heterodimers is formed that allows utilization of different interfaces for distinct functions such as tetramerization (interface B with 6 β-strands only) or binding of cellular targets (interface A with 7 β-strands).

The I72A mutant of A46, which binds lower amounts of fatty acids, indeed showed a reduced ability to inhibit TLR4-stimulated NF-κB-driven transcription compared to the wild-type protein (Fig 5B). The inhibitory level of the wild-type A46 was achieved by the I72A mutant when higher quantities of the mutant protein were expressed. This is not unexpected, as the C-terminal domain alone (A46(87–229)) can bind TIR/TRAM with K_D_ of 3.6 μM (Table 1). Presumably, at higher concentrations, the C-terminus of the A46 I72A mutant can compensate for the loss of binding of the lipid-containing N-terminal domain. Pertinently, we have shown that the C-terminus of A46 alone is capable of efficiently inhibiting MyD88-mediated NF-κB activation when IL-1β stimulation system is used [9]. TLR4-stimulated activation of the NF-κB transcription factor involves both TRAM and MyD88-dependent cascades [36]; taken together, our data suggest that the N-terminal domain of A46 may play a more appreciable role in the inhibition of the TRAM pathway than the MyD88 pathway.

To find similarities of A46(1–83) to other known folds, we searched the PDB database with PDBeFold [37], using subunit A of A46(1–83) as search query. The highest match corresponded to the nuclear movement protein from *E*. *cuniculi* GB-M1 (PDBID 2O30, chain B). Six secondary structure elements were aligned involving 57 residues at an rmsd of 3.39 Å for 45 C_α_; however, the mutual orientation of both sheets is markedly different and the CS domain seen in NudC does not show oligomerisation. Therefore, we searched for similar local folds of the same connectivity using the same core of 45 residues with the program BORGES [25]. The closest match for the strands of ligand bound subunit A was extracted from 2XN2, with 3.09 Å rmsd, whereas for subunit B, a fold extracted from 2OQE gave 2.42 Å. No instance could be identified of an equivalent fold showing the same structural change upon ligand binding; nevertheless, a survey of the hits revealed recurring instances of carbohydrate binding proteins, proteins forming pores and participating in the proper insertion of periplasmic proteins into membranes. These include proteins such as YidC (PDBID 3BLC), located in the periplasmic space of *E*.*coli* that could, theoretically, bind lipidated proteins. The geometry of the local fold described by the 6 sheets is also close to a part found in pore-forming hemolysins and leucodines. Indeed, the S-F heterodimer in the latter ones achieves asymmetry through the association of two components of very different sequence but very close geometry, with up to one C-terminal strand present in only one of the copies [38].

The structure of the A46(1–83) protein illuminates the oligomerization state of both the N-terminal extension and the full-length protein. Previous data had indicated the presence of a tetramer in solution for the full-length A46 and a dimer for the Bcl-2-like C-terminal domain [9]. The structure of the N-terminal extension shows a tetramer formed by the association over a crystallographic twofold axis of the two copies present in the asymmetric unit (S1 Fig), evaluated to be persistent under physiological conditions. SAXS analysis confirmed that that A46(1–83) is tetrameric in solution (Fig 6A).

For full-length A46 in solution, structural information on the separated N- and C-terminal domains allowed interpretation of the envelope generated by SAXS. The full-length molecule has an elongated shape, with the N- and C- domains linked by a flexible, proteolytically sensitive linker that allows movement of the two domains relative to each other (Fig 6C). Rigid body fitting of the structures in the envelope of the full-length A46 using CORAL software [39] indicated a movement of 90 degrees between the two domains.

**Fig 7:**
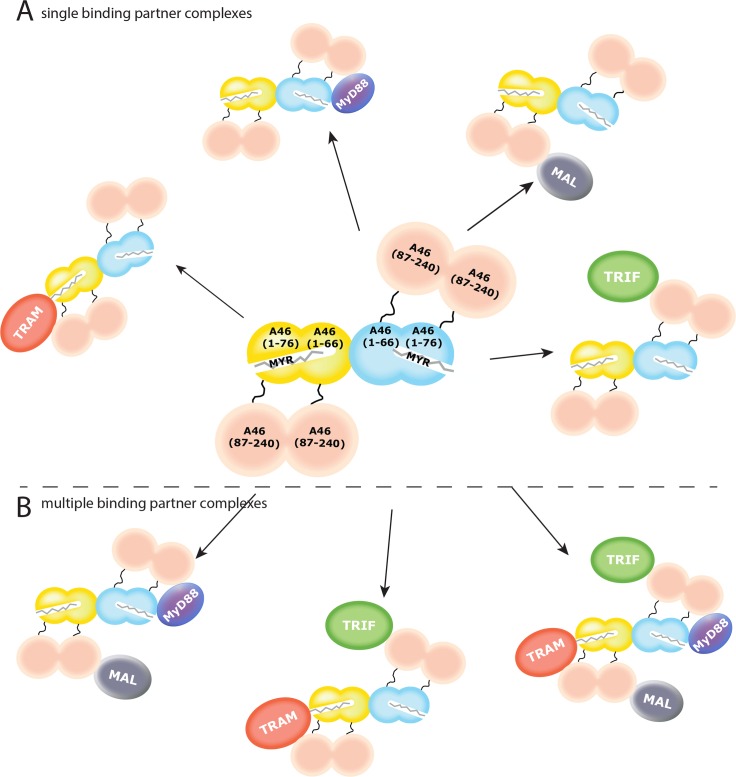
Scheme of the postulated interactions of the full-length tetrameric A46 with the TIR domains of MyD88, MAL, TRIF and with full-length TRAM. The tetramer of A46 is composed of the N-terminal domain (one yellow and one light blue dimer) and C-terminal domain (in salmon). The A subunit of the N-terminal domain A46(1–76) can either bind MyD88 via protein-protein interactions or use its hydrophobic cavity to accommodate the myristate of TRAM. The possible array of interactions can include single protein-protein interactions, such as with MyD88, TRAM, MAL or TRIF alone (A), or simultaneous multiple protein-protein complexes in which some or all TIR adaptor proteins bind to a single tetrameric molecule of A46 (B). TRAM is presented in red and the myristate, either free or post-translational modification of TRAM, is in grey sticks. TIR/MAL is in purple-grey, TIR/MyD88 is in blue-purple, TRIF is in green.

What are the implications of this structural data for the function of A46 in inhibiting signalling through the TRAM and MyD88 linked pathways? We note that both the N-terminal and C-terminal domains of A46 can bind the TIR domains of MyD88 (Fig 7A), although the binding of the N-terminal domain is tenfold lower and the expression of this domain alone does not inhibit IL-1 induced NF-κB mediated signalling in cells (Table 1, Fig 2). However, we suggest that this bipartite binding enables A46 to generate a chain around the TIR domain of MyD88 that would prevent the association of its death domain to assemble the Myddosome, an important structure in the development of the inflammatory response [40]. Additionally, we propose the binding of the myristate post-translational modification of TRAM by the N-terminal domain of A46, with the remainder of the TIR domain of TRAM being bound by the C-terminal Bcl-2 domain of A46 (Fig 7A). For the TIR domain of MAL, an interaction was only observed with the C-terminal domain of A46; the binding site on A46 for the TIR domain of TRIF has not yet been determined. We propose here that the interaction is only with the C-terminal domain (Fig 7A).

The above model assumes binding of only one single TIR domain to the A46 tetramer. However, as depicted in Fig 7B, each tetramer can theoretically present four binding sites for TIR domains. We speculate therefore that A46 could form complexes with multiple binding partners. Indeed, it can even be envisaged that one molecule of A46 could bind one molecule each of MyD88, MAL, TRAM and TRIF (Fig 7B, right side). Thus, even with low initial concentrations of A46, this arrangement would serve to strongly inhibit the inflammatory response by keeping MyD88 death domains apart, preventing proper cellular localization of TRAM and sequestering the other signalling and adaptor molecules. Future experimentation will show the accuracy of these predictions.

## Materials and Methods

### Plasmids

The cloning of the plasmid containing full-length sequence of the A46R gene from the VACV Western Reserve strain plasmids (NCBI Gene ID:3707702) as well as of those encoding TIR domains of mammalian MAL and murine MyD88 was described previously [9]. The N-terminal portion of A46 was amplified from the plasmid containing full-length A46 [9] at different length using following primers for the indicated fragments: F: 5’-CGCAAGCCATGGCACAGCAAATGGCGTTTGATATATC-3’ and R: 5’-GCCCGGATCCTTAACT ATACTTATTATACAAGTAAGTC-3’ for the fragment A46(1–90); F: 5’-CGCAAGCCA TGGCACAGCAAATGGCGTTTGATATATC-3’ and R: 5’-GCCCGGATCCTTAAGTCATACTAA CCGGCGTATTAAC-3’ for the fragment A46(1–83); and F: 5’-CGCAAGCCATGGCACAGCA AATGGCGTTTGATATATC-3’ and R: 5’-GCCCGGATCCTTAACCAATATTAGTTTCCTCTG-3’ for the fragment A46(1–73). Obtained fragments were digested with *Nco*I and *Bam*HI restriction enzymes and ligated into the pET-TRX1 containing HIS_6_-TEV-thioredoxin as an expression tag.

To generate variants of A46(1–83) to examine their lipid-binding properties, we performed PCR mutagenesis using the pTRX-A46(1–83) plasmid as a template and the following primers: F3D, F: 5’-GCAAATGG CGGATGATATATCAG -3’ and R: 5’- CTGATATATCAT CCGCCATTTGC -3’; H36L F: 5’- GTTAATGATACACTCTACACTGTCG -3’ and R: 5’- CGACAGTGTAGAGTGTATCATTAAC -3’; Y37A, F: 5’-GATACACACGCCACTGTCGA-3’ and R: 5’-TCGACAGTGGCGTGTGTATC-3’; Y37W, F: 5’-GATACAC ACTGGACTGTCGAATTTG -3’ and R: 5’-CAAATTCGACAGTCCAGTGTGTATC-3’; I72A, F: 5’- GAAACTAATGCTGGTTGCGCGG -3’ and R: 5’- CCGCGCAACCAGCATT AGTTTC -3’. The generated PCR products were digested with *Dpn*I and subsequently transformed in *E*.*coli* TOP10 competent cells.

For expression in mammalian cells, the plasmids coding for the full-length A46 and C-terminal portion of A46 with the respective tags were cloned previously [9]. For the cloning of the N-terminal domain of A46(1–83), the gene was obtained by amplification from the plasmid carrying the full-length A46 with the primers F: 5’-GCCCGAATTCCGAGAATGGAGCAGAAACTCA TCTCTGAAGAGGATCTGGCGTTTGATATATC-3’ and R1: 5’-CCGCTCGAGTTACTTA TCGTCGTCATCCTTGTAATCAGTCATACTAACCGGCG-3’ or R2: 5’- CCGCTCGAGTTA AGTCATACTAACCGGCG-3’ to yield myc-A46(1–83)-FLAG or myc-A46(1–83), respectively. The amplified DNA fragments were digested with *Xho*I and *Eco*RI restriction enzymes and ligated into the pCAGGS vector [41].

The plasmid encoding a GST-fusion of the TIR domain of human TRAM (amino acid residues 66–235) was a kind gift from Dr. H. Tochio [42].

**Expression and purification of full-length A46**, **TIR/MyD88 and TIR/MAL** were performed as described previously [9].

### Expression and purification of the N-terminal part of A46

*E*. *coli* BL21 (DE3) competent cells were transformed with the plasmids coding for the variants of the N-terminal domain of A46. The expression was performed in 2 liters of LB medium containing kanamycin (50 mg/liter). The cells were grown at 37°C until the mid-log phase (A_600_ = 0.6). Expression was induced with 0.25 mM isopropyl 1-thio-β-D-galactopyranoside at 23°C. After 4 hours, cells were harvested and resuspended in 20 mM Tris-HCl, pH 8.5, 100 mM NaCl, 25 mM imidazole, 5% glycerol and 10 mM β-mercaptoethanol. An EmulsiFlex C3 homogenizer (Avestin) was used for cell lysis. The soluble phase was cleared from insoluble material by centrifugation at 18000 rpm for 30 min. Recombinant proteins were bound to Ni-NTA agarose (5 Prime) charged with 300 mM NiCl_2_ and pre-equilibrated with lysis buffer. Resin was washed with five column volumes of lysis buffer and proteins of interest were eluted in three column volumes of 20 mM Tris-HCl, pH 8.5, 300 mM NaCl, 200 mM imidazole and 15 mM β-mercaptoethanol. Recombinant TEV protease was added to release A46 domains by proteolysis during overnight dialysis against 20 mM Tris-HCl, pH 8.5, 150 mM NaCl, 10 mM imidazole and 15 mM β-mercaptoethanol. The protein of interest was separated from the protease and the tag by four passages through Ni-NTA resin pre-equilibrated with the dialysing buffer. The resulting protein solution was dialysed against 20 mM Tris-HCl, pH 8.5 and 2 mM DTT for 2 hours. SEC with a HiLoad 16/60 Superdex 75 (GE Healthcare) was performed as final purification step in 20 mM Tris-HCl, pH 8.5 and 10 mM DTT. The concentration of the protein of interest was measured by NanoDrop ND-1000 (Thermo Scientific). The accuracy of NanoDrop measurements was confirmed by additional measurement of the concentration of two samples from independent purifications using BCA Protein Assay Reducing Agent Compatible kit (Thermo Scientific) as described by the manufacturer.

### Microscale thermophoresis

Microscale thermophoresis protein-protein interaction studies were performed on the Monolith NT.115 (Nanotemper Technologies, Munich) using fluorescently labeled proteins as described [43, 44]. For the TIR/MAL, TIR/MyD88 and TIR/TRAM protein labeling, the standard labeling kit for the fluorescent dye Alexa Fluor 647 from Nanotemper was used. Solutions of unlabelled A46(1–229), A46(87–229) and A46(1–83) were serially diluted from 150–450 μM to 8–20 nM in the presence of 30–70 nM of one of the labeled TIR/MAL, TIR/MyD88 or TIR/TRAM proteins. Measurements were performed at 25°C in 20 mM TrisHCl pH 8.5, 100 mM NaCl, 5% glycerol, 1 mM TCEP, 1 mM EDTA, 0.05% Tween 20 using 50% LED power and 60% or 80% IR-laser power. Data analysis was performed with Nanotemper analysis software, v.1.2.101.

### Protein crystallisation and data collection

Crystals of A46(1–83) and A46(1–73) were initially obtained at protein concentration of 6.75 and 3.5 mg/ml, respectively, in 20 mM TrisHCl pH 8.5 and 10 mM DTT in multiple buffer formulations of the PACT Premier crystallization screen (Molecular Dimensions, Suffolk, UK) using the sitting-drop vapour diffusion technique and a nanodrop-dispensing robot (Phoenix RE; Rigaku Europe, Kent, United Kingdom). We obtained crystals of both protein constructs; however, for A46(1–73) the crystals were not amenable for diffraction experiments. For A46(1–83), the largest crystals grown in 100 mM HEPES 7.0, 20% PEG6000 and 0.2 M of one of following salts NaCl, LiCl or NH_4_Cl were mounted in the loop and flash-cooled in liquid nitrogen. Crystals with selenium methionine labeled A46(1–83) were obtained in the same buffer formulations. The diffraction data set was collected at 100K at the peak of Se at λ = 0.979 Å at the beamline MASSIF-1 ID30A-1 at the European Synchrotron Radiation Facility (Grenoble, France) to 1.55 Å resolution and processed using the XDS package [45]. Crystals belonged to the space group C2 (a = 65.79 Å b = 59.5 Å c = 47.26 Å).

### Structure determination and refinement

The structure was solved by ARCIMBOLDO_BORGES *ab initio* phasing software [25] combining fragment search with Phaser [26] and density modification with SHELXE [46] on the supercomputer Gordon at the SDSC.

Autobuilding was carried out using the program AutoBuild from the Phenix package [47]. The structure was refined using the program Phenix Refine [48] and manual adjustments with the software Coot [49]. Stereo-chemistry and structure quality were checked using the program MolProbity [50]. Data collection and refinement statistics are reported in Table 2.

### Accession code

The coordinates of the A46(1–83) X-ray structure have been deposited in the Protein Data Bank (PDB) database, accession number 5EZU. The experimental SAXS data and derived models of the either full-length A46 or its N-terminal domain have been deposited in small angle scattering biological data bank (SASBDB) with the deposition codes SASDBL7 and SASDBK7.

### SAXS analysis

SAXS experiments for the A46(1–83) and full-length A46(1–229) were performed at 0.9918 Å wavelength ESRF at BioSAXS beamline BM29 coupled to the Superdex 200 10/300 exclusion column (Grenoble, France) and equipped with PILATUS 1M detector at 2.867 m distance from the sample, 0.04 < q < 0.5 Å-1 (q = 4π sin θ/λ, 2θ is the scattering angle). The data were collected using protein concentrations of 15.5 and 4.4 mg/ml for the A46(1–83) and A46(1–240), respectively. The samples were in a buffer containing 20 mM Tris-HCl pH 8.5, 10mM DTT and the measurements were performed at 20°C. The data were processed and analyzed using the ATSAS program package [51]. The radius of gyration R_g_ and forward scattering I(0) were calculated by Guinier approximation. The maximum particle dimension D_max_ and P(r) function were evaluated using the program GNOM [52]. To demonstrate the absence of concentration dependent aggregation and interparticle interference in the both SAXS experiments, we inspected Rg over the elution peaks and performed our analysis only on a selection of frames in which Rg was most stable (S4 Fig). Overall, such stability of Rg over the range of concentrations observed in the SEC elution indicates that there were no concentration-dependent effects or interparticle interference. The data collection and structural parameter from SAXS analysis are summarized in Table 3. The *ab initio* models were derived using DAMMIF [53]. 40 individual models were created for each run, which were then overlaid and averaged using DAMAVER. For the oligomeric state assessment, the theoretical scattering from either theoretical dimer or tetramer using the high-resolution structure (5EZU) was performed. First, the residues missing in the crystal structure were added by CORAL modelling; later the theoretical scattering curves were generated using CRYSOL and compared to the SAXS experimental data for A46(1–83). To obtain a pseudo-atomic model of the full-length A46, CORAL [39] software was used with the structures for A46(1–83) (5EZU) connected by dummy residue linkers to A46(87–229) (4LQK); the C-terminal domain A46(87–229) was extended by 16–19 dummy residues to imitate the full length of the A46 protein.

**Cell culture and reporter gene assays** were performed as reported previously [9]. The following expression plasmids were used: the full-length myc-A46(1–240)-FLAG (amounts 200, 150 ng), the N-terminal domain A46(1–83)-FLAG (100, 50 ng), the C-terminal domain A46(87–229)-FLAG (400, 300 ng). The amount of DNA per well was kept constant at 500 ng by supplementation with pCAGGS empty vector.

Human embryonic kidney cells 293 stably transfected with TLR4 or MD2 were kind gifts from Dr. Sylvia Knapp. HEK293-TLR4 and HEK293-MD2 were maintained in DMEM supplemented with 10% fetal calf serum, 1% penicillin/streptomycin and 0.5 mg/ml geniticin G418. To perform a reporter assay with a wild-type or lipid-binding mutant of A46, HEK293-TLR4 cells were grown in 24-well plates and transfected with 80 ng pNF-κB-luc reporter plasmid (Firefly luciferase), 20 ng of pRL-TK (*Renilla* luciferase) internal control and 300 ng of the respective A46 containing plasmid. The supernatant from HEK293-MD2 cells was filtered and added in a ratio of 1:4 with DMEM to HEK293-TLR4 in the stimulation assay. 40 hours post transfection, HEK293-TLR4 cells were stimulated by addition of MD-2 supernatant, DMEM and 500 ng/ml of LPS. After 7 h, cells were collected, lysed in Passive Lysis Buffer (Promega) and whole cell lysates were analyzed for luciferase activity using the Dual-Luciferase Reporter Assay (Promega). Firefly luciferase activity was normalized by *Renilla* luciferase activity.

Expression levels of myc-A46-FLAG and myc-A46-FLAG I72A in HEK293T cells were estimated by western blotting. Myc-tagged A46 variants were detected with monoclonal anti-myc 4A6 antibody at a dilution of 1:1000 (Millipore), ϒ-tubulin was used as a loading control and detected with monoclonal anti-tubulin GTU-88 antibody at a 1:5000 dilution (Sigma).

### Analysis of fatty acid content by negative ion ES-MS survey scans

Lipid extractions from purified recombinant proteins were achieved by two different methods. Method A: 3 successive vigorous extractions with 10 volumes of diethyl ether after treatment with 2 volumes of 6M HCl overnight. The ether extracts were evaporated under nitrogen and analysed by electrospray mass spectrometric and tatty acid methyl ester analysis as described below.

Method B: 3 successive vigorous extractions with ethanol to fully denature proteins (final 90% v/v) [54]. The pooled extracts were dried by nitrogen gas in a glass vial and analysed by electrospray mass spectrometry.

For electrospray mass spectrometry analysis, extracts were analyzed on a Absceix 4000 QTrap, a triple quadrupole mass spectrometer equipped with a nanoelectrospray source as described previously [55].

Quantification of the fatty acids from method A were done by conversion to the corresponding fatty acid methyl esters (FAME) followed by GC-MS analysis as described previously [56] using the following GC temperature program: 70°C for 12 min followed by a gradient to 220°C at 4°C/min and held at 220°C for a further 10 min. Mass spectra were acquired from 50–500 amu. The identity of FAMEs was carried out by comparison of the retention time and fragmentation pattern with mixtures of FAME standards.

## Supporting Information

S1 Fig Tetramer as a functional state of A46(1–83). A, Stereo view of crystal packing of A46(1–83). The tetramer is composed of two symmetry related dimers, one in yellow and the other in blue. B, Comparison of A46(1–83 the dimer (A/B) and the tetramer (B/B) interfaces. Dimers are presented in yellow and blue, residues involved in interface formation are in sticks, comprising eight salt bridges, 27 H-bonds and numerous hydrophobic bonds (S2 Table). In the B/B tetrameric interface, the PISA server finds 10 salt bridges, 20 H-bonds and hydrophobic bonds (S3 Table). Insets show enlargement of dimer and tetramer interfaces. Drawings were made using PyMOL [29].
(TIF)

S2 Fig The tunnel is shown in white on the sagittal cut of the A46(1–83) dimer, rotated to adjust orientation to that of the graph. The radius values and the length of the tunnel are presented in the graph. The star indicates the starting point of the tunnel. Measurements and visualization were performed with online software MOLE 2.0 (http://mole.upol.cz/) [30, 31].
(TIF)

S3 Fig Omit difference electron density map for myristic acid accommodated in the cavity of A46(1–83) dimer. The map is contoured at 1σ and calculated with coefficients |F_o_−F_c_|. The myristate is presented in sticks, where carbon atoms are blue and oxygen ones are red.
(TIF)

S4 Fig Analysis of Rg over elution peak in SEC-SAXS. The Rg (black curve) and I(0) (red curve) are plotted versus recorded frames in SEC-SAXS profiles for either A46(1–83) or A46(1–240) proteins. The frames used for the further analysis and model building are highlighted in gray. For A46(1–83), frames 354–389 were used; for A46(1–240), frames 479–499 were used.
(TIF)

S1 Table Relative quantification of the major fatty acids extracted from A46(1–83) variants. (DOCX)

S2 Table Residues involved in formation of dimeric interfaces. (DOCX)

S3 Table Residues involved in formation of tetrameric interfaces. (DOCX)
