# Supplementary figures and images for: Vaccinia Virus Immunomodulator A46: A Lipid and Protein-Binding Scaffold for Sequestering Host TIR-Domain Proteins
Source: PLoS Pathog. 2016 Dec 14;12(12):e1006079. doi: 10.1371/journal.ppat.1006079 (PMC5156371; doi:10.1371/journal.ppat.1006079)

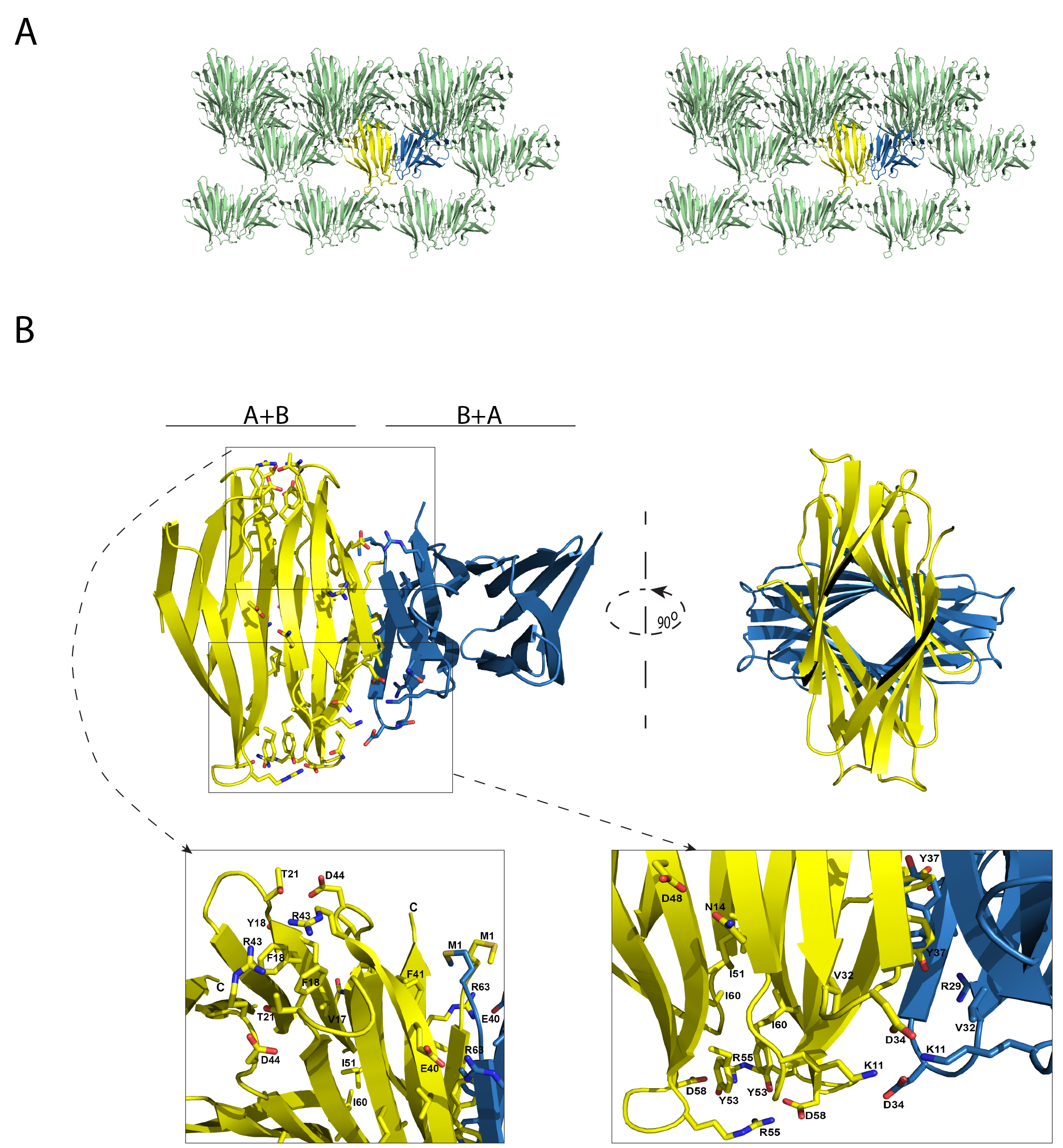

Supplement: S1 Fig — A, Stereo view of crystal packing of A46(1–83). The tetramer is composed of two symmetry related dimers, one in yellow and the other in blue. B, Comparison of A46(1–83 the dimer (A/B) and the tetramer (B/B) interfaces. Dimers are presented in yellow and blue, residues involved in interface formation are in sticks, comprising eight salt bridges, 27 H-bonds and numerous hydrophobic bonds (S2 Table). In the B/B tetrameric interface, the PISA server finds 10 salt bridges, 20 H-bonds and hydrophobic bonds (S3 Table). Insets show enlargement of dimer and tetramer interfaces. Drawings were made using PyMOL [29]. (TIF) [file ppat.1006079.s001.tif]

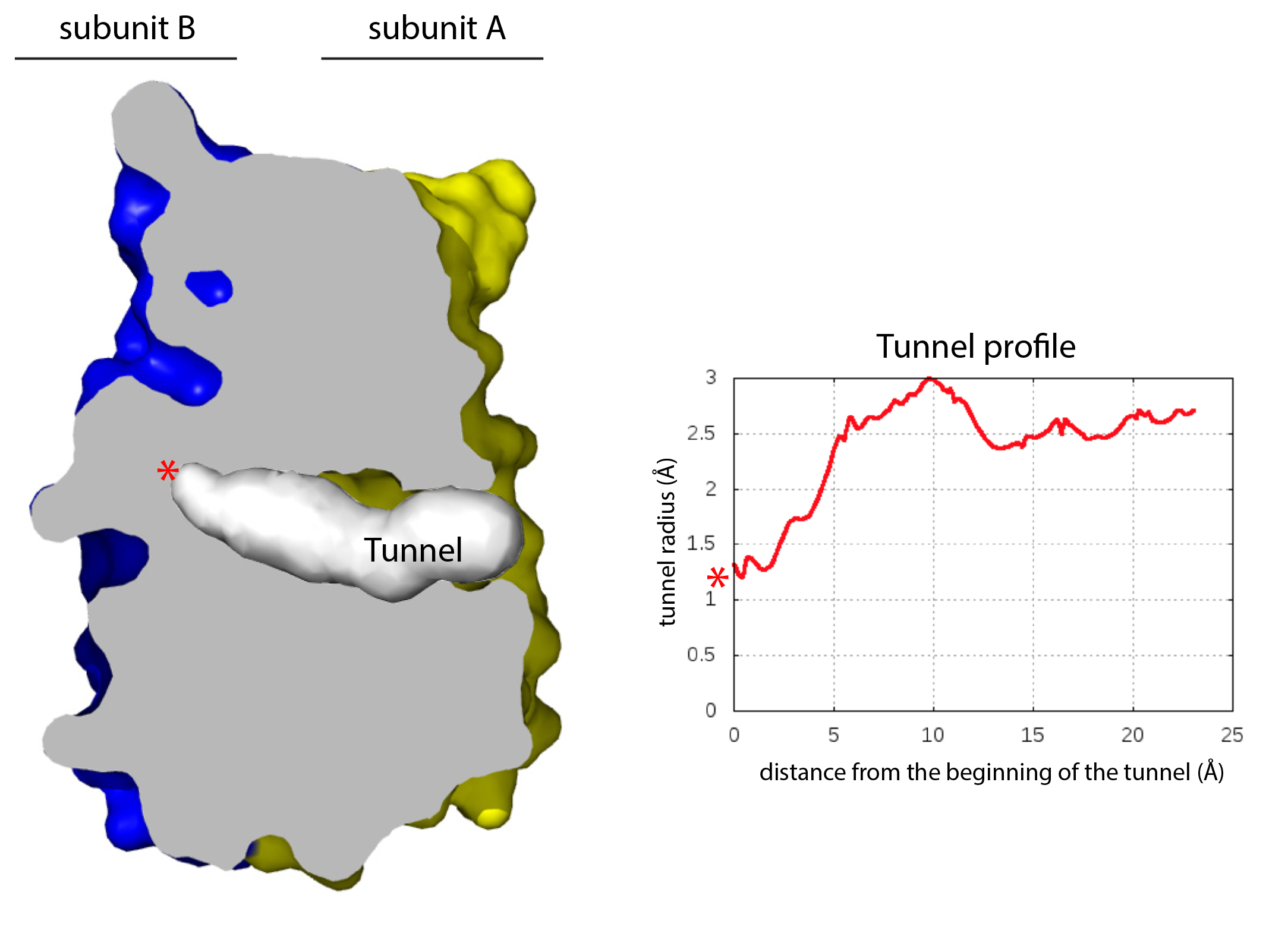

Supplement: S2 Fig — The radius values and the length of the tunnel are presented in the graph. The star indicates the starting point of the tunnel. Measurements and visualization were performed with online software MOLE 2.0 (http://mole.upol.cz/) [30, 31]. (TIF) [file ppat.1006079.s002.tif]

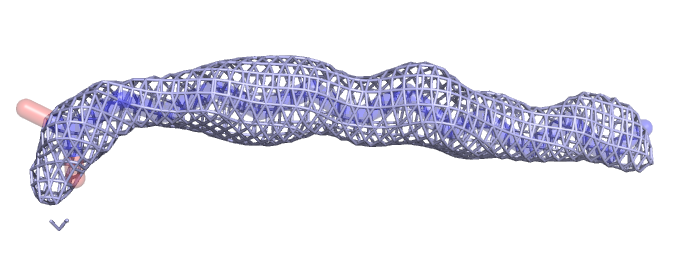

Supplement: S3 Fig — The map is contoured at 1σ and calculated with coefficients |Fo−Fc|. The myristate is presented in sticks, where carbon atoms are blue and oxygen ones are red. (TIF) [file ppat.1006079.s003.tif]

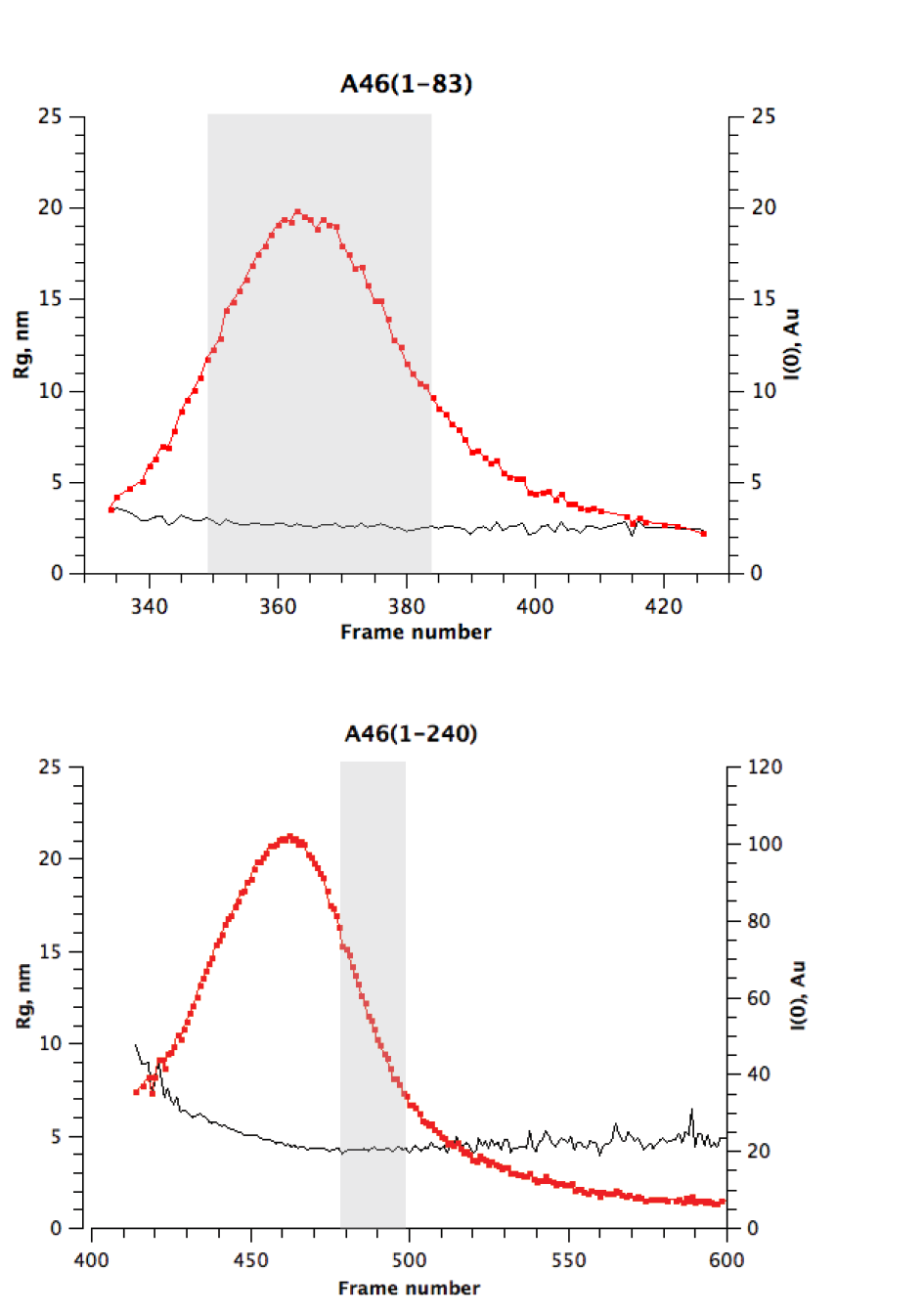

Supplement: S4 Fig — The Rg (black curve) and I(0) (red curve) are plotted versus recorded frames in SEC-SAXS profiles for either A46(1–83) or A46(1–240) proteins. The frames used for the further analysis and model building are highlighted in gray. For A46(1–83), frames 354–389 were used; for A46(1–240), frames 479–499 were used. (TIF) [file ppat.1006079.s004.tif]
