# Supplementary material for: Vaccinia Virus Immunomodulator A46: A Lipid and Protein-Binding Scaffold for Sequestering Host TIR-Domain Proteins
Source: PLoS Pathog. 2016 Dec 14;12(12):e1006079. doi: 10.1371/journal.ppat.1006079 (PMC5156371; doi:10.1371/journal.ppat.1006079)
Supplement: S1 Table — (DOCX) [file ppat.1006079.s005.docx]

Table S1. Relative quantification of the major fatty acids extracted from A46(1-83) variants.

Values obtained from FAME extraction and subsequent GC-MS analysis were normalized to the wild-type (100%) by the amounts of protein extracted and an internal standard of 20 μl of 10 mM C24:0.

| Mutation | Relative content of selected fatty acids | | | Relative total amount of fatty acids (%) |
| --- | --- | --- | --- | --- |
|  | C14:0 | C16:1 | C16:0 |  |
|  |  |  |  |  |
| Wild-type | 44,7 | 5,5 | 49,8 | 100 |
| F3D | 11,4 | 12,2 | 16,5 | 40,1 |
| H36L | 16 | 25,1 | 12 | 53,1 |
| Y37A | 29,8 | 34,7 | 27,3 | 91,8 |
| Y37W | 11,1 | 16,8 | 13,1 | 41,0 |
| I72A | 8,4 | 12,8 | 7,4 | 28,6 |
