# Supplementary material for: Vaccinia Virus Immunomodulator A46: A Lipid and Protein-Binding Scaffold for Sequestering Host TIR-Domain Proteins
Source: PLoS Pathog. 2016 Dec 14;12(12):e1006079. doi: 10.1371/journal.ppat.1006079 (PMC5156371; doi:10.1371/journal.ppat.1006079)
Supplement: S2 Table — (DOCX) [file ppat.1006079.s006.docx]

Table S2. Residues involved in formation of dimeric interfaces

| Residue | Accessible surface area, Å2 | Buried surface area, Å2 | Length of bond, Å | Bond type | Interacting residue |
| --- | --- | --- | --- | --- | --- |
| Dimeric interface (A/B) | | | | | |
| Asn8 | 66.12 | 0.27 |  | Hph |  |
| Ala9 | 30.04 | 27.35 |  | Hph |  |
| Ser10 | 104.01 | 18.84 | 2.90 | H | Arg55 |
| Lys11 | 102.40 | 2.08 |  | Hph |  |
| Thr12 | 112.95 | 52.94 |  | Hph |  |
| Ile13* | 27.28 | 27.28 | 2.93/2.82 | H | Ile51 |
| Asn14 | 74.25 | 49.86 |  | Hph |  |
| Ala15* | 55.72 | 41.16 | 2.88 | H | Thr49 |
| Leu16 | 73.21 | 40.50 |  | Hph |  |
| Val17* | 99.11 | 79.96 | 3.03/3.01 | H | Val47 |
| Tyr18 | 98.22 | 48.93 |  | Hph |  |
| Phe19* | 84.06 | 84.06 | 2.84 | H | Asp44 |
| Ser20 | 18.16 | 6.29 |  | Hph |  |
| Thr21* | 120.56 | 53.82 | 2.94/2.38/2.88 | H | Arg43/Asp44 |
| Gln22 | 175.24 | 0.59 |  | Hph |  |
| Leu26 | 38.83 | 38.67 |  | Hph |  |
| Ile28 | 17.01 | 7.34 |  | Hph |  |
| Phe41 | 0.32 | 0.32 |  | Hph |  |
| Asp42 | 45.55 | 0.12 |  | Hph |  |
| Arg43 | 144.20 | 78.52 | 3.21 | H | Thr21 |
| Asp44 | 124.11 | 32.88 | 3.88/3.02 | H | Thr21 |
| Lys45 | 98.04 | 11.85 |  | Hph |  |
| Val46 | 110.64 | 51.70 |  | Hph |  |
| Val47* | 27.52 | 27.52 | 2.87 | H | Val17 |
| Asp48 | 59.92 | 32.82 | 2.86 | H | Asp14 |
| Thr49* | 58.47 | 40.79 | 2.73/2.83/2.83/2.79 | H | Ala15/Asp14 |
| Phe50 | 112.40 | 38.97 |  | Hph |  |
| Ile51* | 127.83 | 106.11 | 2.93/2.82 | H | Ile13 |
| Ser52 | 58.17 | 30.07 |  | Hph |  |
| Tyr53 | 104.65 | 76.63 |  | Hph |  |
| Arg55 | 201.16 | 83.74 | 2.92/3.32/3.38/2.81 | HS | Asp58 |
| Asp58 | 61.51 | 21.92 | 3.02/3.51/3.53/2.88 | HS | Arg55 |
| Ile60 | 46.10 | 46.10 |  | Hph |  |
| Ile62 | 16.07 | 6.87 |  | Hph |  |
| Pro67 | 120.49 | 3.69 |  | Hph |  |

*main chain H-bonding
