# Supplementary material for: Vaccinia Virus Immunomodulator A46: A Lipid and Protein-Binding Scaffold for Sequestering Host TIR-Domain Proteins
Source: PLoS Pathog. 2016 Dec 14;12(12):e1006079. doi: 10.1371/journal.ppat.1006079 (PMC5156371; doi:10.1371/journal.ppat.1006079)
Supplement: S3 Table — (DOCX) [file ppat.1006079.s007.docx]

Table S3. Residues involved in formation of tetrameric interfaces

| Residue | | Accessible surface area, Å2 | | Buried surface area, Å2 | | Length of bond, Å | Bond type | | Interacting residue | | |
| --- | --- | --- | --- | --- | --- | --- | --- | --- | --- | --- | --- |
| Tetrameric interface (B/B) | | | | | | | | | | | |
| Met1 | 194.18 | | 151.23 | |  | | | Hph | |  |  |
| Ala2* | 57.78 | | 42.52 | | 3.07/2.92 | | | H | | Glu40 |  |
| Phe3 | 14.73 | | 9.52 | |  | | | Hph | |  |  |
| Asp4* | 75.53 | | 43.77 | | 2.88/2.79 | | | H | | Thr38 |  |
| Ile5 | 18.39 | | 15.77 | |  | | | Hph | |  |  |
| Ser6* | 62.59 | | 51.35 | | 2.98/2.93 | | | H | | His36 |  |
| Val7 | 15.45 | | 15.37 | |  | | | Hph | |  |  |
| Asn8* | 69.61 | | 30.84 | | 3.64 | | | H | | Asp34 |  |
| Lys11 | 102.40 | | 39.79 | | 3.28 | | | HS | | Asp34 |  |
| Lys25 | 58.31 | | 10.87 | |  | | | Hph | |  |  |
| Arg29 | 76.37 | | 3.64 | |  | | | Hph | |  |  |
| Asp34 | 116.76 | | 57.96 | | 3.64/3.28 | | | H,HS | | Asn8/Lys11 |  |
| Thr35 | 84.41 | | 71.75 | |  | | | Hph | |  |  |
| His36* | 96.25 | | 56.40 | | 2.93, 2.98 | | | H | | Ser6 |  |
| Tyr37 | 92.74 | | 83.26 | | 3.34 | | | H | | Thr38 |  |
| Thr38* | 50.63 | | 39.82 | | 2.79/2.88/3.34 | | | H | | Asp4/Tyr37 |  |
| Val39 | 25.52 | | 19.16 | |  | | | Hph | |  |  |
| Glu40* | 98.33 | | 83.57 | | 2.92/3.84/3.63/2.33/3.46 | | | HS | | Arg63/Ala2 |  |
| Phe41 | 28.00 | | 16.44 | |  | | | Hph | |  |  |
| Asp42 | 69.19 | | 14.09 | |  | | | Hph | |  |  |
| Lys45 | 98.04 | | 13.82 | |  | | | Hph | |  |  |
| Arg63 | 109.28 | | 27.01 | | 3.63/2.33/3.84/2.33/3.63 | | | HS | | Glu40 |  |
| Leu66 | 51.41 | | 7.18 | |  | | |  | |  |  |
|  |  | |  | |  | | |  | |  |  |

*main chain H-bonding
